# Defining a Uterine Extraction Score based on a Volume/Access Ratio in Total Hysterectomy: a retrospective cohort study

**DOI:** 10.52054/FVVO.16.1.009

**Published:** 2024-03-28

**Authors:** S Schoenen, L de Landsheere

**Affiliations:** Department of Obstetrics and Gynecology, Hôpital de la Citadelle, University of Liège (ULiège), 4000 Liège, Belgium

**Keywords:** Hysterectomy, Uterine extraction score, Morcellation, Laparoscopy, Laparotomy

## Abstract

**Background:**

Regardless of the technique used, extraction of the uterus is a crucial step in hysterectomy. There is currently no scoring system to predict its feasibility.

**Objectives:**

Our main objective was to determine a predictive score of uterine extraction feasibility to optimise surgical planning of total hysterectomy. As secondary objectives, we examined the correlation between uterine volume predicted by preoperative ultrasound and the final weight of the surgical specimen and analysed the impact of the uterine extraction modality on operative and hospitalisation times.

**Materials and Methods:**

We defined a Uterine Extraction Score (UES) based on the ratio between uterine sizes and vaginal access. This score was retrospectively applied to a cohort of 178 patients who were hysterectomised for benign conditions between January 2019 and December 2022.

**Main outcome measures:**

The UES allows identification of three groups of decreasing feasibility of vaginal extraction, symbolised by traffic light colours: green - vaginal extraction without morcellation, orange - vaginal extraction with morcellation, red - abdominal morcellation by mini-laparotomy or primary laparotomy.

**Results:**

The results show that the UES-predicted, and the observed routes of extraction concord in 92% of cases. There is a strong correlation between estimated volume and final uterine weight. Uterine morcellation lengthens the operative time and the hospital stay.

**Conclusions:**

The UES seems to be a reliable tool to predict the route of uterine extraction in total hysterectomy.

**What is new?:**

The development of a new scoring system empowers surgeons with decisive information to enhance perioperative outcomes.

## Introduction

Hysterectomy is one of the main interventions in gynaecological surgery. Historically, laparotomy and vaginal routes were gold standards until the advent of minimally invasive surgery. Harry Reich performed the first laparoscopic hysterectomy in Pennsylvania in 1988 in the US (Sutton, 1997). The technique has since evolved with the development of robot-assisted systems and the emergence of Vaginal-Natural Orifice Transluminal Endoscopic Surgery (v-NOTES) (Housmans et al., 2020; Raquet et al., 2023). Regardless of the technique used, extraction of the uterus is a crucial step in the procedure. Tissue morcellation can be required in the case of a large uterus. Different morcellation routes are available: laparoscopic, vaginal, or abdominal by mini-laparotomy (Meurs et al., 2017; Clark and Cohen, 2018). In April 2014, the U.S. Food and Drug Administration (FDA) warned surgeons about the risk of free intraperitoneal uterine morcellation that may disseminate cells of undiagnosed cancer in the abdominal cavity (Xu et al., 2021; FDA, 2014). Unconfined morcellation can also cause non-malignant sequelae, such as ectopic myoma, iatrogenic endometriosis or disseminated peritoneal leiomyomatosis (Tulandi et al., 2016). The morcellation procedure requires therefore a specific equipment, i.e. a safety bag (Moawad et al., 2016a).

There is currently no scoring system to predict the feasibility of uterine extraction. Such a system would be of great clinical utility, helping to choose the most adequate and safe surgical strategy. Based on literature data and our clinical experience, we have devised a Uterine Extraction Score (UES) that can be easily used in clinical practice and has the potential to guide surgical decisions for uterine extraction in hysterectomy.

## Materials and Methods

We developed a predictive score of the feasibility of uterine extraction and its route based on uterine size and vaginal accessibility. The size of the uterus, coded by letters, was evaluated retrospectively according to weight and volume (Table I): (A) volume ≤ 250cm3 or weight < 250g, (B) 250- 500cm3 or 250-500g, (C) >500cm3 or >500g and (D) >1000cm3 or >1000g.

**Table I:** Uterus size and vaginal accessibility are the two variables of the UES score. Uterus size is estimated according to the ultrasound uterine volume measurement and confirmed by the uterine weight on pathological analysis. Vaginal accessibility is classified according to the patient’ parity.

Uterus size	Vaginal accessibility
A < 250cm - 250g	0 = no vaginal delivery
B = 250-500cm^3^ - 250-500g	1 = one vaginal delivery
C = 500-1000cm^3^ - 500g	2 if > 1 vaginal delivery
D > 1000cm - 1000g	

We applied the formula of the Morphological Uterus Sonographic Assessment (MUSA) group (Van den Bosch et al., 2015): (d1 (total length - cervix) x d2 (anteroposterior diameter) x d3 (transverse diameter) x 0,523) to predict uterine volume. When ultrasound data were not available, we use pathological specimen measurements. The correlation between the uterine volume estimated by ultrasound and final weight was analysed with Pearson’s Correlation Coefficient.

Vaginal accessibility was scored according to the number of vaginal deliveries (Table I): 0 if the patient had no vaginal delivery, 1 if she had one, and 2 for more than one vaginal delivery.

We defined three composites of “traffic light” colour scores susceptible to predicting the surgical outcome of uterine extraction: green for vaginal extraction without morcellation, orange for vaginal extraction with morcellation, red for abdominal extraction by mini-laparotomy or laparotomy. As shown in Table II, we assigned the size/accessibility scores to the colour score according to the predominant extraction route in the respective categories:

**Table II:** Uterine Extraction Score based on the ratio between uterine size and vaginal access, identified by traffic light colours: green -vaginal extraction without morcellation possible, orange -vaginal extraction but morcellation needed, red -abdominal morcellation by mini-laparotomy or primary laparotomy required. Results of the retrospective application of the score to 178 patients who were hysterectomised for benign condition.

Access			
Size	0	1	2
A	34/34 = 100%	28/29 = 97%	66/66 = 100%
B	5/10 = 50%	2/3 = 67%	13/16 = 81%
C	6/7 = 86%	1/1 = 100%	3/5 = 60%
D	0	2/2 = 100%	1/2 = 50%

- Scores 0A - 1A - 2A - 2B are assigned to green, indicating that vaginal extraction without morcellation would be feasible.

- Scores 1B - 1C - 2C - 2D are assigned to orange, suggesting that vaginal extraction would be feasible if uterine morcellation is performed.

- Scores 0B - 0C - 0D - 1D are assigned to red, meaning that laparoscopic hysterectomy with abdominal morcellation by mini-laparotomy or primary laparotomy is needed.

We retrospectively applied the UES to 178 patients who underwent a hysterectomy for benign conditions between 2019 to 2022. To avoid a potential operator-related bias, we selected patients who were treated by the same senior surgeon (LdL) with experience in minimally invasive surgery. Exclusion criteria were supra-cervical hysterectomy or hysterectomy on suspicion of neoplasia. Patients’ characteristics were collected from their electronic medical records. Operative time was calculated between entrance to and exit from the operating room.

The main objective of the study was to explore the potential predictive value of the UES for uterine surgical extraction. The project was approved by the ethics committee of the University Hospital of Liège (B4122022000031).

## Results

**Figure 1:**
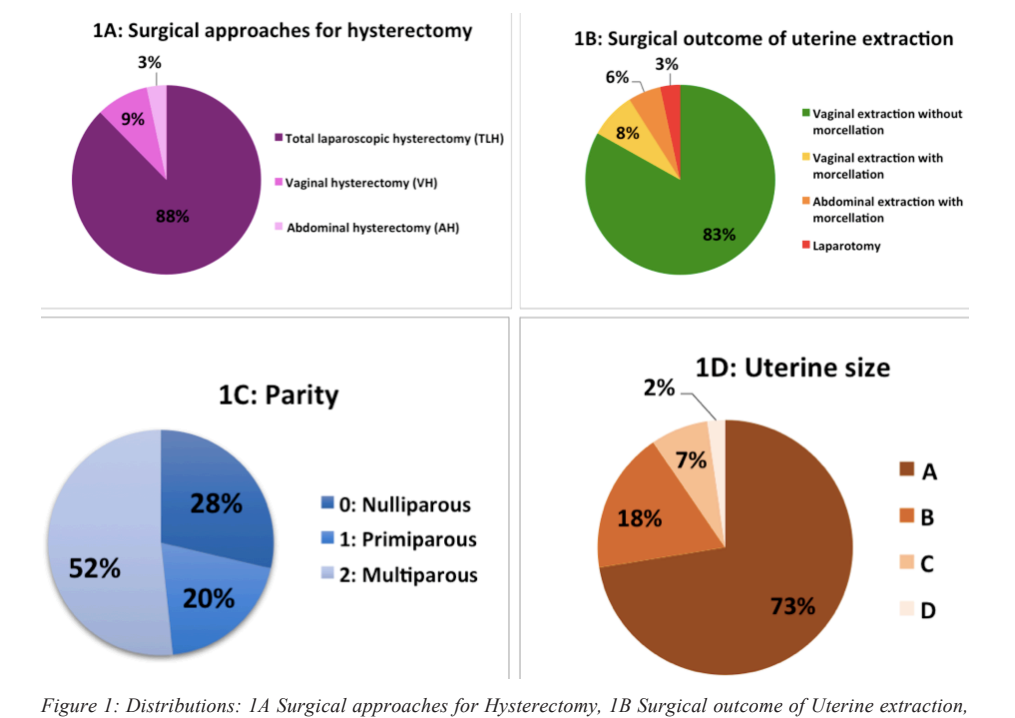
Distributions: 1A Surgical approaches for Hysterectomy, 1B Surgical outcome of Uterine extraction, 1C Parity, and 1D Uterine size.

A total of 178 patients who underwent hysterectomy for benign conditions were included in the study. Hysterectomy was performed by laparoscopic surgery (n=156, 87,6%), by vaginal approach (n=16, 9%), or by laparotomy (n=6, 3,4%) (Figure 1A). Uterine extraction was performed vaginally without morcellation (n=148, 83%), vaginally with morcellation (n=14, 8%), by mini- laparotomy for abdominal morcellation (n=10, 6%) or by laparotomy (n=6, 3%) (Figure 1B). When morcellation was needed (n=24, 14%), it was performed vaginally (n=14, 58%) or by mini- laparotomy (n=10, 42%). Uterus morcellation was always performed using a safety bag. There was no case of laparoscopic morcellation. The median operative time was 65 minutes (±23 SD), and the median hospital stay was 2 days (±0,5 SD).

Parity used to determine the vaginal accessibility was distributed as follows (Figure 1C): 51 patients had no vaginal delivery (29%), 35 had one vaginal delivery (20%) and 92 had more than one vaginal delivery (52%). Uterine sizes evaluated by the final weight of the operative specimen and uterine volume were classified A in 129 (72%), B in 32 (18%), C in 13 (7%), and D in 4 cases (2%) (Figure 1D).

**Figure 2:**
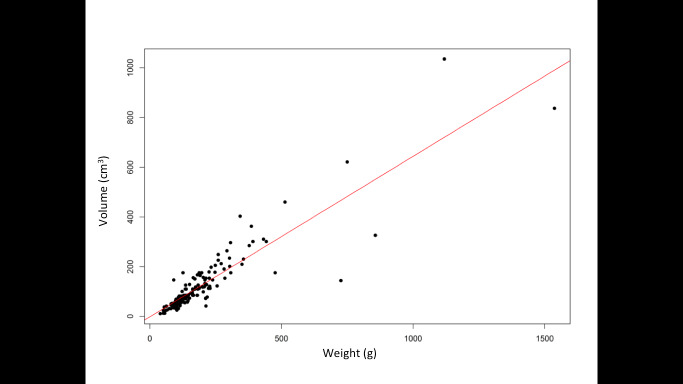
Correlation curve between estimated uterine volume and final weight (Pearson Correlation Coefficients r=0,9; P<,0001).

There was a strong correlation between preoperative estimated uterine volume and final weight (Pearson Correlation Coefficients r=0,9; P<,0001; Figure 2).

Based on these data, we computed retrospectively the UES for each patient and evaluated the concordance rate between the surgical outcome predicted by the UES and the effective operative uterine extraction method. The results are summarised in Table II.

Patients scored 0A, 1A, 2A and 2B had a predicted “green” surgical outcome, i.e. vaginal extraction without morcellation, which was confirmed in 84% to 100% of the cases.

Among 10 patients with a 0B score (red), 4 had abdominal extraction with morcellation and 1 a laparotomy, whereas 2 had vaginal extraction without morcellation and 3 with morcellation, i.e. a 50% concordance rate.

Among 3 patients classified 1B (orange), 2 had a vaginal extraction with morcellation while 1 underwent vaginal extraction without morcellation, i.e. a 67% concordance rate. Of 19 patients with a 2B score (green), 16 had vaginal extraction without morcellation and 3 had vaginal extraction with morcellation (84% concordance).

Concordance was 86% in 7 patients classified 0C (red): 3 had laparoscopic hysterectomy with abdominal morcellation, 3 laparotomy, and 1 vaginal extraction with morcellation. One patient classified 1C underwent vaginal extraction with morcellation (100% concordance).

Among patients with a 2C score (orange), 3 had vaginal extraction with morcellation, 1 vaginal extraction without morcellation and 1 abdominal extraction for bulky myoma, which means a 60% correct prediction.

No patient was classified 0D.

Two patients classified 1D (red) had abdominal extraction (total concordance).

One out of 2 patients classified as 2D had vaginal extraction with morcellation, the other had laparotomy for fibroma of more than 10cm (50% concordance).

The need of morcellation lengthens the operative time by 40 minutes on average (P<.0001) and the hospital stay by 1 day (P=.0002).

Patients’ characteristics according to the surgical outcome are found in the Table III. No significant difference in body mass index (BMI) was found according to the need of morcellation.

**Table III:** Patients’ characteristics according to the surgical outcome of uterine extraction. The results are considered significant at the 5% uncertainty level (P-value <0.05).

Variables	Vaginal extraction n=148 Mean ± SD Number (%)	Vaginal morcellation n=14 Mean ± SD Number (%)	Abdominal extraction n=16 Mean ± SD Number (%)	P-value
Age (years)	45.7 ± 6.35	47.7 ± 6.28	49.8 ± 6.65	0.014
Menopause status	7 (4.7)	1 (7.1)	3 (18.8)	0.056
BMI (kg/m^2^)	26.1 ± 4.22	26.9 ± 6.92	27.1 ± 6.50	0.36
Vaginal accessibility				0.0072
0	36 (24,3)	4 (28,6)	11 (68,8)	
1	29 (19,6)	3 (21,4)	3 (18,8)	
2	83 (56,1)	7 (50)	2 (12,5)	
Uterus size				<.0001
A	36 (24,3)	4 (28,6)	1 (6,3)	
B	29 (19,6)	3 (21,4)	5 (31,3)	
C	83 (56,1)	6 (42,9)	7 (43,8)	
D	0	1 (14,3)	3 (18,8)	
Operative time (min)	65.1 ± 16.0	104 ± 25.6	98.6 ± 32.3	<.0001
Hospital stay (days)	2.28 ± 0.51	2.64 ± 0.63	2.75 ± 0.58	0.0002

Four perioperative complications were reported: two vaginal haematomas and one vaginal abscess in the vaginal extraction group without morcellation.

One of the mini-laparotomy cases developed a haematoma at the sub-pubic incision during the hospitalisation stay, requiring reoperation.

## Discussion

### Main Findings

We defined a composite score based on vaginal accessibility and uterine volume (“UES-Uterine Extraction Score”) to predict the feasibility of uterine extraction during total hysterectomy. We applied the UES in a retrospective analysis of 178 hysterectomised patients and found that it would have reliably predicted the surgical procedure in 92% of patients (κ concordance coefficient 0,73). In addition, we found that uterine morcellation lengthens operative time and hospital stay (P<.0001), independently of the morcellation route.

### Interpretation

A clinical score able to guide the strategic decision about the operative difficulty and the route of uterine extraction for total hysterectomy is not available in the literature. To predict the surgical outcomes of hysterectomies, Uccella et al. (2021) proposed a “Large Uterus Classification” when the uterine fundus is at or over the transverse umbilical line. Details on morcellation and uterine extraction, however, are not available in this study. We propose a score focused on the feasibility of uterine extraction. As suggested by our retrospective chart review, it would have been able to predict the uterus extraction outcome in 92% of hysterectomies.

One of the factors in the UES is vaginal accessibility deduced from the number of vaginal deliveries. Admittedly, this is a rather simple way of assessing vaginal access that does not depend only on the number of vaginal deliveries but also on other critical factors like the weight of the newborn, instrumentation during delivery, etc. However, this score should be easy to compute during routine consultation and not depend on too many factors. In any case, the surgeon has to take into account the clinical gynaecological examination, an item that could be included in the score in a future prospective study.

The 0B group is heterogeneous with a concordance rate of 50% with the predicted route of abdominal morcellation or laparotomy. Only 10% of this group had vaginal extraction without morcellation. The remaining 90% needed morcellation, 50% by mini- laparotomy, and 50% by vaginal approach.

There was no significant difference in BMI between the 3 prognostic groups (green, orange, red). Minimally invasive surgery is recommended for obese patients (Tyan et al., 2020). Operative times and complications increase with higher BMI (Davidson et al., 2022; Mikhail et al., 2015). Regarding the morcellation route, a prospective study showed a lower BMI in the vaginal cohort, but no clear recommendation was found in the literature (Cohen et al., 2019).

Our results show that the need for morcellation lengthens operative time and hospital stay. If applied in clinical practice, the UES could thus allow for better planning of the surgical strategy: optimise the operating schedule, prepare the appropriate materials (morcellator, morcellation bag), and choose the most appropriate anaesthesia. Abdominal extraction and laparotomy are indeed painful for the patient. Anticipating the procedure allows the anaesthesiologist to plan post-operative analgesia e.g. to perform a transversus abdominal plan (TAP) block to minimise post-operative pains (Mathew et al., 2019).

In our cohort, the most common surgical procedure was laparoscopy, which is similar to the national data obtained by the Belgian National Institute for Health and Disability Insurance (NIHDI) between 2008 and 2021 regarding the surgical routes for hysterectomy. Trends in laparoscopic surgery are changing with a growing part for robotic-assisted hysterectomy. Vaginal morcellation is less commonly used in robotic surgery than in conventional laparoscopic surgery because of the limited vaginal access (Shashoua et al., 2009). Vaginal accessibility could, however, be improved by changing the position of the legs and undocking the robot.

Uterine volume, the second factor in the UES, was estimated retrospectively according to the Morphological Uterus Sonographic Assessment (MUSA) group’s formula using measurements of the pathological specimens when pre-operative ultrasound data were not available. There was a strong significant correlation between the estimated volume and the final weight (r = 0,9 - P<.0001). These results have to be confirmed by a prospective study, in which all uterine volumes should be measured pre-operatively by ultrasound.

Recent data shows that endoscopic power morcellation in containment bags is safe and limits tissue dissemination (Clark and Cohen, 2018). Contained manual morcellation, however, has similar outcomes than power morcellation, but a shorter operation time and significantly better cost- effectiveness (Güven and Uysal, 2023). This is the reason why we perform only contained manual morcellation in our institution, either by a vaginal or by a mini-laparotomy approach, depending on the clinical evaluation. All specimens are enclosed in an endoscopic bag under visual control: Alexis Containment Extraction System (Applied Medical, Rancho Santa Margarita, CA) or Endocatch bag (Covidien, Mansfield, MA). They are thereafter morcellated extracorporeally with a scalpel blade using the “Paper Roll” uterine morcellation technique (Wong et al., 2010; Moawad et al., 2016a). The vaginal and mini-laparotomy approaches have similar outcomes (Cohen et al., 2019). Several authors report an increased risk of pelvis infection during vaginal morcellation (Moawad et al., 2016b). Extraction through colpotomy incision is preferred after total hysterectomy if vaginal access allows it. In case of nulliparous patient or small pelvic dimensions, a mini-laparotomy is performed by enlarging the umbilical or supra-pubic incision. In our survey, there were 3 complications in the vaginal extraction group, but they were not related to morcellation. Finally, vaginal extraction could increase bag leakage during morcellation (Cohen et al., 2019; Solima et al., 2015). Although no leakage tests were performed in our study, there were no reports of tissue spillage after morcellation in safety bags

### Strengths and Limitations

The major strength of our study is to propose a clinical uterine extraction score that is easily applicable in routine practice and has an excellent predictive value of 92%.

The main limitation of our study is its retrospective monocentric design and the relatively small number of patients, especially for large uterine sizes (scores C and D). As mentioned above, other limitations are that vaginal access was evaluated solely on number of deliveries. The traffic light colours were assigned retrospectively according to the most frequent ways of uterine extraction. We plan, therefore, to validate our results in a larger, multicentre prospective study, taking into account these limitations.

## Conclusions

Based on a retrospective chart review, the Uterine Extraction Score (UES) seems to be a reliable tool to predict the feasibility, ease, and route of uterine extraction during total hysterectomy for benign conditions. It could therefore help the surgeon to anticipate the difficulty of the procedure and its impact on operative time and hospital stay, which needs to be confirmed in a larger prospective study.
